# Work and work exposures in sugarcane farming in Eswatini, Southern Africa

**DOI:** 10.1007/s00420-025-02140-z

**Published:** 2025-05-14

**Authors:** S. C. Msibi, S. Naidoo, K. Jakobsson, J. Glaser, B. Skinner, R. N. Naidoo

**Affiliations:** 1 Discipline of Public Health Medicine, School of Nursing and Public Health, University of KwaZulu-Natal, Durban, South Africa; 2 Discipline of Public Health Management, Institute of Development Management, Manzini, Eswatini; 3 Discipline of Occupational and Environmental Health, School of Nursing and Public Health, University of KwaZulu-Natal, Durban, South Africa; 4 School of Public Health and Community Medicine, Institute of Medicine, Sahlgrenska Academy, University of Gothenburg, 405 30 Gothenburg, Sweden; 5 La Isla Network, 2219 California Ave NW, #52, Washington, DC, 20008 USA; 6 School of Sport, Exercise and Rehabilitation Sciences, University of Birmingham, Birmingham, UK

**Keywords:** Eswatini, Heat stress, Sugarcane worker, Workload, Wet bulb globe temperature (WBGT)

## Abstract

**Objective:**

To describe work practices and exposures among sugarcane farm workers on smallholder cooperatives in eSwatini, being subcontracted (cane cutters) or directly employed (pesticide applicators).

**Methods:**

Data were collected at mid-harvest using repeated field observations and wet bulb globe temperature (WBGT) measurements. Questionnaires were administered to 267 sugarcane cutters and 125 pesticide applicators. Individual work output was defined as the length of the row of sugarcane cut over the workday, which also determined the monthly remuneration. The Quick Exposure Check was used to assess exposure to musculoskeletal risks. Pesticide handling practices were described with a focus on personal protection safety practices. Additionally, heart rate was measured in 20 pesticide workers for estimation of workload and core temperature.

**Results:**

Sugarcane sites generally had no provision for rest in shade. Few workers were provided with drinking water and thus used personal containers or took water from the irrigation system. The mean water intake over the workday was as low as 1.4 L. Most workers (87%) described their work as physically demanding. For workers with a high workload (cane cutters), the observed daily average maximum WBGT of 28.6 °C was above the NIOSH recommended exposure limit (REL) of 26 °C. Pesticide applicators had a moderate workload. A minority of the applicators had access to proper personal protective equipment such as air respirators (4%), chemical gloves (17%), and chemical overalls (21%); still, their protective clothing hindered heat dissipation and thus increased heat stress.

**Conclusion:**

Workplace interventions are needed to protect workers' health and safety.

**Supplementary Information:**

The online version contains supplementary material available at 10.1007/s00420-025-02140-z.

## Introduction

The sugarcane farming sector is one of the significant sources of employment and livelihood support systems for communities in many countries worldwide (Cockburn et al. 2014; Kaahwa et al. 2023; Medina Hidalgo et al. 2024). However, as in many agricultural sectors, there are challenges for workers, which include prolonged exposure to high levels of ambient heat, which in combination with a heavy workload can lead to heat stress (Lucas et al. 2023) and resulting ill health. Especially, acute and chronic kidney injuries have been repeatedly reported (Bodin et al. 2016; Crowe et al. 2023; Dally et al. 2018; Glaser et al. 2020). The physically demanding work may cause musculoskeletal injuries (Phajan et al. 2014; Ruths et al. 2023). Additionally, there is the risk of chemical exposure from pesticide application during crop maintenance (Lari et al. 2023; Staudacher et al. 2020). These varied practices and exposures demonstrate the demanding and often hazardous nature of working in the sugarcane farming environment (Ruths et al. 2023).

When workers are exposed to high ambient heat, the metabolic work rate, clothing, and lack of hydration will further increase the heat stress (Lucas et al. 2023). The impacts of heat stress in occupational settings include significant loss of productivity and increased risks for diseases and injuries (Bernard 2014). Workers in agriculture are particularly at risk for heat-related illness (Gibb et al. 2024) and may further be the most impacted, as the working conditions often are characterized by a high degree of informality and lack of occupational health and safety measures (Kjellström et al. 2019).

Sugarcane is an important commodity crop in many tropical and subtropical regions, including Southern Africa, and is heavily dependent on the use of pesticides. With mean annual temperatures already rising, and projected to increase on average between 1.2 °C and 3.3 °C in the Sub-Saharan region by the end of the twenty-first century (IPCC 2022), the cane crops are vulnerable to the effects of climatic extremes engendered by climate change, with increasing water deficits and declining crop yields (Ngcobo et al. 2023). Similarly, the agricultural workforce is vulnerable, and preventive measures are needed to protect the workers from the risk of heat stress. This will also be beneficial for productivity (Amoadu et al. 2023; Hansson et al. 2024b; Radir et al. 2017).

Several studies addressing work environment, heat stress, and health in the sugarcane industry have been done, especially in Central America, Brazil and Asia (Bodin et al. 2016; Boonruksa et al. 2020; Dally et al. 2018; Glaser et al. 2022; Hansson et al. 2024a; Kiatkitroj et al. 2021; Santos et al. 2015) In contrast, only limited information exists from sub-Saharan Africa (Ekiti et al. 2018; H. Hathaway et al. 2023).

Notably, published research from the sugarcane industry has hitherto come from large sugar mills with directly employed workforces (Boonruksa et al. 2020; Glaser et al. 2020; Sorensen et al. 2020). In contrast, the present study, aims to describe the workforce and the work situation on smallholder cooperative farms in Sub-Saharan Africa with informal work arrangements, subcontracting, and unclear or absent occupational safety and health responsibility. This situation is common in many sugar-producing economies.

## Materials and methods

### Setting and design

We conducted a cross-sectional study among sugarcane workers in Eswatini in September 2022. Eswatini is a small landlocked country in Southern Africa, with an area of about 17,360 km^2^ and a population of about 1.1 million. Sugarcane production is the foundation of the agricultural economy, accounting for more than half of the country's annual agricultural output and approximately 8% of the gross domestic product (Nhamo 2017). Sugarcane is grown in low-lying, high-humidity areas with temperatures reaching 40 °C (Tourism et al. 2011). El Niño-induced drought and extreme heat events occurred in 2015–16 and again in 2023–24, increasing the risk of heat-related adverse health outcomes for sugarcane workers in the country's already hottest regions (Ainembabazi 2018; Varotsos et al. 2024).

The total area of sugarcane, reported by all organized groups of associations and large mills growing sugarcane in Eswatini, is about 60,000 hectares (ESA 2023). Our study area is located in the sugar belt region in the Lowveld (lowlands) (Nhamo 2017), characterized by annual average maximum temperatures close to 33 °C (Eswatini 2022). The area comprised 23 sites belonging to the Komati Downstream Development Project Farmers Federation (KDDPFF), representing the majority of organized agricultural associations involved in sugarcane, horticulture, maize, and livestock farming (ESNAU 2023). Among the agricultural cooperative groups in Eswatini, KDDPFF is the largest cooperative involved in sugarcane production, with a membership of 24 farmer associations.

### Study source population

Based on records from the KDDPFF administration office, the number of workers on their sugarcane farms during the study period was 1,879, averaging 77 per association. These comprised 1,020 general laborers, 430 sugarcane cutters, 304 weeding workers, and 125 pesticide applicators. This study focused on the sugarcane cutters, known to have a high physical workload, and the pesticide applicators, all males.

#### Sugarcane cutters

Sugarcane cutters are seasonal contracted workers who work annually during the harvest season from around the first week of May to the end of October or the first week of November. The cutters are recruited by subcontractors and organized into harvesting groups, each providing cutting services to a specific group of sugarcane farmers' associations. Each harvesting group consists of two to four companies, with an estimated average of twenty workers per company. The subcontractor is responsible for contractual arrangements with the farmers’ associations, with their remuneration based on the tonnage of sugarcane cut over the harvest. The subcontractor provides the field supervisors for the companies.

The sugarcane cutters are a mix of Eswatini nationals, who comprise the majority, and migrant workers from neighbouring countries. Migrant workers live in temporary camps while local workers live in their homes. At the end of the harvesting season, migrant workers usually return to their home countries, and Eswatini nationals disperse to their home areas or continue scouting for other temporary informal employment such as bricklaying, welding, and security guarding.

#### Pesticide applicators

Pesticide applicators are either contracted for the season or permanently employed by a specific farmer's association, usually two to ten persons per association. While most of the farmer associations employ pesticide applicators solely for pesticide work, there may be instances where some applicators perform additional tasks, such as weeding and applying fertilizer. Pesticide applicators are usually residents, living close to the sugarcane fields; therefore, they usually walk or use bicycles to and from work.

### Recruitment of study participants

The KDDPFF administration office provided a list of 430 sugarcane cutters and 125 pesticide applicators from which the sample was extracted. Based on available resources, only 400 workers could be included. Because of their smaller number, all pesticide applicators were included. The remaining 275 of the sample size were then allocated to sugarcane cutters, randomly selected from the full list. All selected workers were male; no women were employed as cutters or pesticide applicators.

The study protocol included observations, questionnaires, and biological measurements. Workers were informed about the purposes of the study and any risks before they signed consent forms. None of the workers refused to participate.

### Timing of data collection

The months between August and December in Eswatini have the highest temperatures (Supplementary Fig. 1). September was selected as the best period to collect data during the harvest. September marks the mid-harvest and is at the end of the winter and the beginning of the spring season, where temperatures begin to rise. The chances of rainfall are still low, reducing the impact on fieldwork. It was also assumed that workers would be acclimatized to the demands of their tasks by mid-harvest. For logistical reasons, the workplace visits for structured assessments of musculoskeletal workload and measurements of heart rate took place in mid-November.

#### Workplace observations

During the study's conceptualization, the principal investigator (SM) visited the study area before developing the study protocol to observe the fields, locations, and work arrangements over 3 days. At a later stage, in advance of data collection, the principal investigator and a research assistant spent 5 days visiting the study sites to inform the sugarcane cutters and pesticide applicators about the study. The meetings also made interacting with the workers' supervisors possible. Data collection activities began a week later, and the 9 days of primary data collection also provided opportunities for work observations. Additionally, 3 days of workplace visits took place in November 2022.

#### Questionnaire

Ten trained research assistants conducted face-to-face interviews using a structured questionnaire (see Supplement) developed by the principal investigator based on a literature review and consultations with experts (SN, RN, and KJ). The questionnaire included information on demographics (age, sex, language, nationality); general information (medical history, use of non-steroidal anti-inflammatory drugs; alcohol, energy drinks and soda intake, water intake at home); and the current occupation (current job, number of days/week working, number of days/week doing physically demanding work, water intake at work, pesticides used).

All participants were asked about pesticide exposure, including job tasks and frequency, personal protection, and application methods. A catalogue of pesticide pictures based on common local agricultural use was shown to easily identify pesticides they had used since they started working in the sugarcane industry. Other pesticides that were not part of the catalogue were also recorded. Pesticides were classified according to the World Health Organization's (WHO) recommended hazard classification (WHO 2020).

The questionnaire was piloted in July 2022, using 12 sugarcane cutters from a farmer association in the Dvokolwako area, away from the actual study area. The questionnaire was translated into the local SiSwati language and back-translated into English. Migrant workers who understood neither the local language nor English were interviewed in a language of their choice with the help of a fellow worker fluent in the chosen language and SiSwati. The interviews were conducted during the shift and lasted about 15 min per participant.

#### Environmental heat exposure monitoring

The Wet Bulb Globe Temperature (WBGT) was measured to estimate environmental heat exposure (Supplementary material 2). An AZ87786 WBGT data logger (AZ®) was placed for one day in nine different sites, selected to measure representative ongoing operations in the area. All farm sites where worker assessments were performed on a particular day were located within a radius of 7.74 km from the location of the data logger and at an altitude range of about 270 to 360 m above sea level. Data was recorded from the beginning of each shift until all study participants had completed the assigned task for the day, with measurements recorded every 10 min.

The assessment of the worker´s heat stress was based on WBGT monitoring data on the day of assessment of work load, using guidelines for acclimatized workers from the National Institute for Occupational Safety and Health (NIOSH 2016) The recommended exposure limit (REL) were 25.0 °C for very heavy workload, 26.0 °C for heavy workload and 28.0 °C for moderate workload (Supplementary material 3).

#### Assessment of workload, physically demanding working days, and work output

A combination of several approaches was used to evaluate workload. The first approach was based on the Quick Exposure Check (QEC) (Oliv et al. 2019) and involved structured observations during one day by a team of twelve observers who participated in a one-week course on measuring workload in occupational settings. Four observers were assigned to three independent groups, and then simultaneously each group of four assessed a group of sugarcane cutters and a group of pesticide applicators. Sugarcane cutters were observed first, around 0800 h for about 45 min, then pesticide applicators 1 h later, for about 30 min. In addition, one cutter and one pesticide applicator who were randomly selected from the two categories of sugarcane workers answered the QEC self-report questions on manual handling of weight, time spent on tasks, amount of force exerted using hands, visual demands of tasks, vibration, and level of difficulty in keeping up with their tasks. By design, the QEC enables the estimation of work-related exposure levels for body postures, repetitive movements, force/load, and task duration for different regions of the body (back, shoulder/arm, wrist/hand, and neck), driving, vibration, work pace and stress (David et al. 2008). Each observer group's scoring was categorised according to the QEC scoring matrix as low, moderate, high, or very high (Supplementary material 4). The results collected by each observer group were used to produce an average score. In addition, the observers also qualitatively described how the sugarcane cutters and pesticide applicators performed their tasks (Oliv et al. 2019).

The next approach was an expert evaluation. A researcher with solid experience of continuous heart rate (HR) measurements in sugarcane workers in Central America (BS) evaluated the work tasks during the field observations. Finally, each worker was asked about the number of perceived physically demanding workdays per week by the question, "On a scale of 0 – 7 (representative of the number of physically demanding workdays in a week), how often do you do physically demanding tasks?".

The length of cane rows cut by each sugarcane cutter during the day of investigation was used to describe the work output. The field supervisor measured the distance cut at the end of the shift with a measuring wheel. This was the standard way to define the payment to the individual workers.

#### Assessment of heat stress and heat strain in pesticide workers

To better understand heat strain risk in pesticide applicators wearing impermeable personal protective equipment (PPE) hindering heat dissipation, physiological workload assessments were conducted in a convenience sample of 20 male workers (35 ± 5 yrs). Assessments were made over three work shifts in November 2022, with each worker assessed once.

WBGT was measured throughout the work shift, using a Kestrel 5400 Heat Stress Tracker (Nielsen-Kellerman Co., Boothwyn, PA, USA). Pesticide applicators were equipped with a Polar® HR monitor (Verity Sense, Polar Electro, Kempele, Finland) immediately before putting on PPE, and collected from workers once spraying activities had ended. As such, collected data does not account for any job tasks outside of pesticide application (e.g., loading/unloading equipment at the mill). HR data was sampled at 0.1-s intervals, then exported and aggregated into 1-min averages (R Core Team, 2023). HR outliers (e.g., abnormal recordings > 220 beats/minute) were removed.

HR data were expressed as a percentage of maximal HR (%HR_max_), with a regression equation used to predict HR_max_ 208 – (0.7 × age) (Tanaka et al. 2001). Physiological workload was categorized based on HR_max_ as: maximal (91–100%); very hard (81–90%); hard (71–80%); moderate (61–70%); light moderate (51–60%); and light (≤ 50%) (Lucas et al. 2023).

Sequential HR measurements were used to calculate estimated core temperature (ECTemp) (Buller et al. 2013) as an indicator of heat strain (i.e., the effect of heat stress on the body) (Flouris et al. 2018). The time spent at an ECTemp ≥ 38 °C was calculated to reflect international occupational health and safety standards, which recommend avoiding core body temperature ≥ 38 °C (ACGIH 2007; Coco et al. 2016).

### Data analysis

Eight participants were removed during data cleaning due to missing sociodemographic and other key data. Thus, the final sample entails 267 sugarcane cutters and 125 pesticide applicators. Descriptive statistics were presented separately for sugarcane cutters and pesticide applicators.

### Ethical clearance

The Eswatini Health and Human Research Review Board (EHHRRB) (EHHRRB075/2021) and the Biomedical Research Ethics Committee (BREC) of the University of KwaZulu-Natal (BREC/00002995/2021) approved the study protocol. Study participants received and signed declaration of consent forms written in English and the local SiSwati language.

## Results

### Working practices for sugarcane cutters

In the afternoon of the day before cutting, the target fields were burned in preparation for cutting. Sugarcane cutters mostly arrived in the fields from 0530 h to start work at approximately 0600 h. In one harvesting group, some workers began before sunrise, as early as 0400 h, to avoid the heat from the sun. The subcontractors were responsible for transporting the sugarcane cutters from hostel camps and residential areas by small trucks in groups of 15 to 20. For some groups, the trucks stayed until their set time to leave the fields, while others returned later to fetch the workers.

**Fig. 1:**
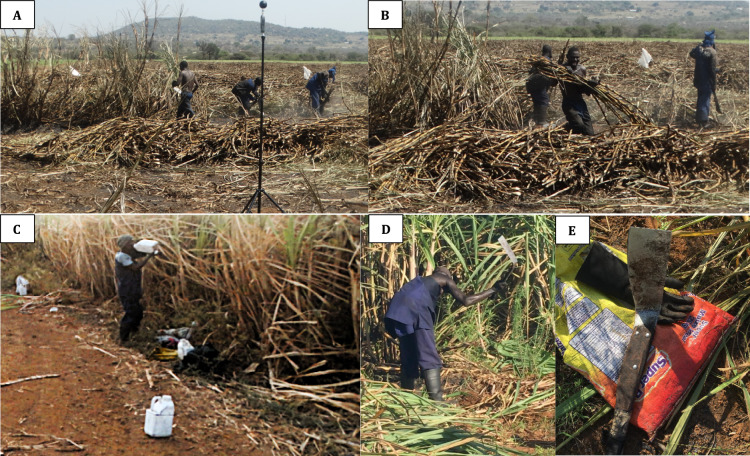
**A** and **B** Sugarcane cutters working; **C** A sugarcane cutter drinking his maize drink from a used pesticide container. Around the sugarcane cutters are containers for his co-workers; **D** Sugarcane cutter working without protection, and **E** working equipment (carrying bag, cutting knife, and glove)

A large part of the first hour of a day's shift was spent assigning sugarcane lines to be cut to the individual cutters. After cutting with handheld knives, workers piled the cane in windrows in readiness for mechanized loading by tractors. Generally, the cutters wore gumboots, long cotton trousers, long-sleeved upper-body cotton clothing, and sunhats, and some had goggles and hand gloves. Due to high temperatures, some sugarcane cutters removed clothing on their upper body to cool themselves (Fig. 1D).

There were no fixed rest periods during the day, but short breaks of about 5 min were individually taken after about 45 min of continuous cutting with different paces of speed. There was no shade where the sugarcane cutters could rest, hence they sat anywhere in the fields, particularly where they placed their drinking containers or at points along the irrigation system where a valve would allow them to collect water. During the breaks, some workers would drink water from containers they brought themselves or from outlet valves of the sprinkler irrigation system. The drinking water brought to the fields came from surface water (40%), tap water (54%), and boreholes (6%). Most sugarcane cutters also brought maize drinks kept in used pesticide containers with removed labels (Fig. 1C). Water from the irrigation system or brought to the field by the workers was also used for handwashing. There were no sanitation stations close to the fields.

The cutters generally exerted themselves to finish cutting their allocated portion of the work. Some cutters finished their tasks as early as 0900 h, while others finished around 1300 h, working an average duration ranging between 3 and 7 h per shift. The distance cut determined the monthly amount of remuneration.

### Working practices for pesticide applicators

Pesticide applicators arrived at the fields around 0530 h to begin their shift around 0600 h. The applicators were usually residents living close to sugarcane fields, who walked or used bicycles for transportation. Pesticides were generally applied in the early morning and only on days when the wind was calm, thus avoiding too windy and high-temperature conditions.

Generally, the working time ranged between 2 and 4.5 h. The daily shift thus ended between 0800 and 1030 h. In most sites, the end of the pesticide application marked the completion of the daily assigned task, but the workday could sometimes be extended with other work tasks. All pesticide applicators were paid at the end of the month based on the number of days worked.

**Fig. 2:**
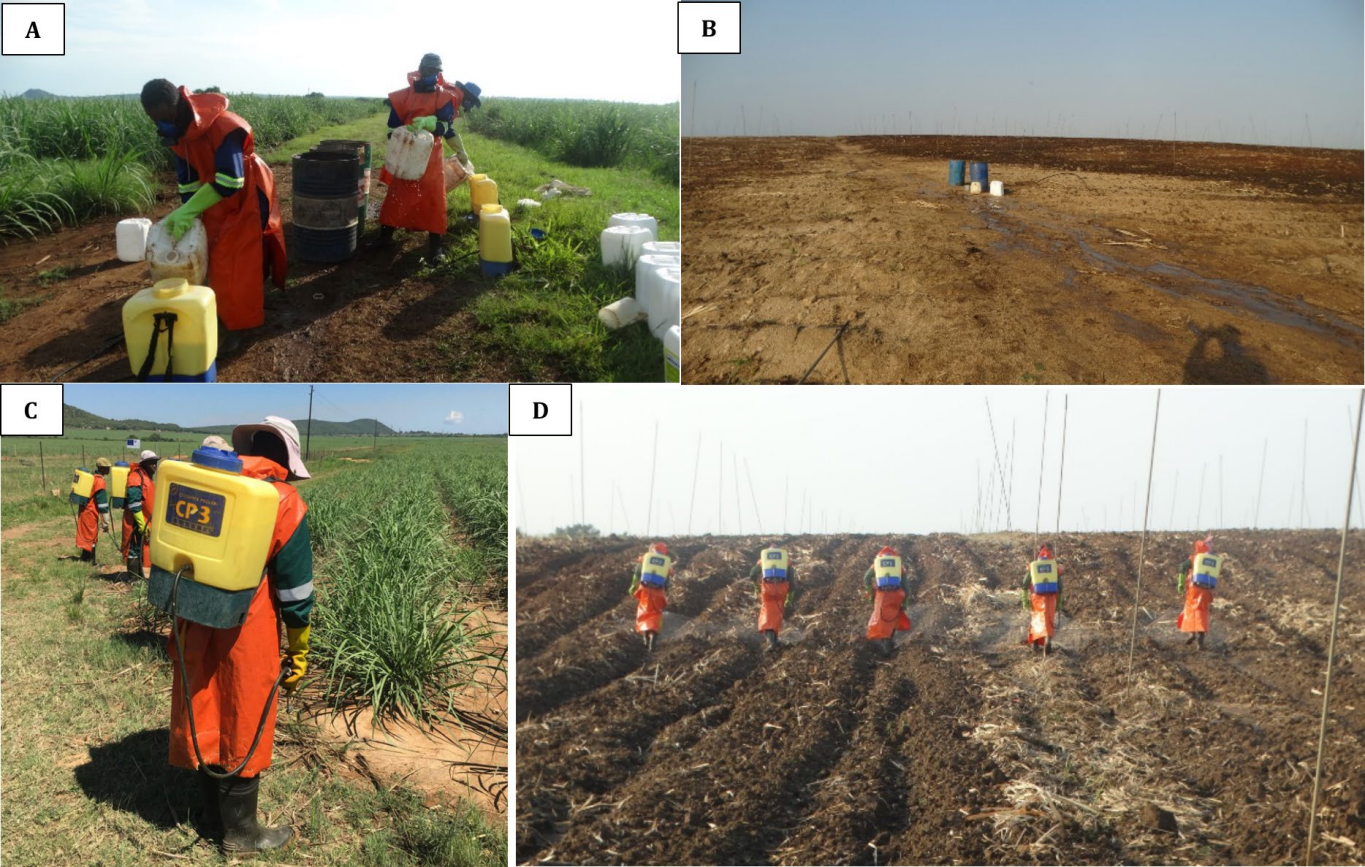
**A** Pesticide applicators mixing pesticide solution; **B** Pesticide mixing station; **C** and **D** Pesticide applicators applying pesticide in sugarcane fields

After mixing the pesticides and preparing the equipment, the pesticides were applied using backpack sprayers with hand-held pumps and nozzles (Fig. 2C, D). The employer provided the workers with rubber boots, two-piece plastic or cloth overalls, plastic aprons, chemical gloves, single-cartridge respirators, goggles, and sunhats. There was also provision of water in all the associations, which the pesticide applicators used for washing their equipment. During spraying, applicators were separated from each other by a row of sugar cane (∼1 m apart; Fig. 2D). Thus, applicators was not only exposed to their own spraying aerosol but also from colleagues nearby.

Pesticide applicators did not have formal breaks but could rest for about 5–10 min when their backpacks were refilled with 15–20 L of pesticide every 15–20 min. No shade or toilet facilities were provided. Water for preparing pesticide solutions was sourced from the irrigation system or storage tanks, transferred to 210 L drums. This water was also used for handwashing and drinking. Reported sources of workplace drinking water were surface water (63%), tap water (30%), and boreholes (7%). Notably, applicators did not use empty pesticide containers for drinking water at work. Unlike the cutters, applicators generally did not eat or consume fluids other than water.

### Wet bulb globe temperature measurements

**Fig. 3:**
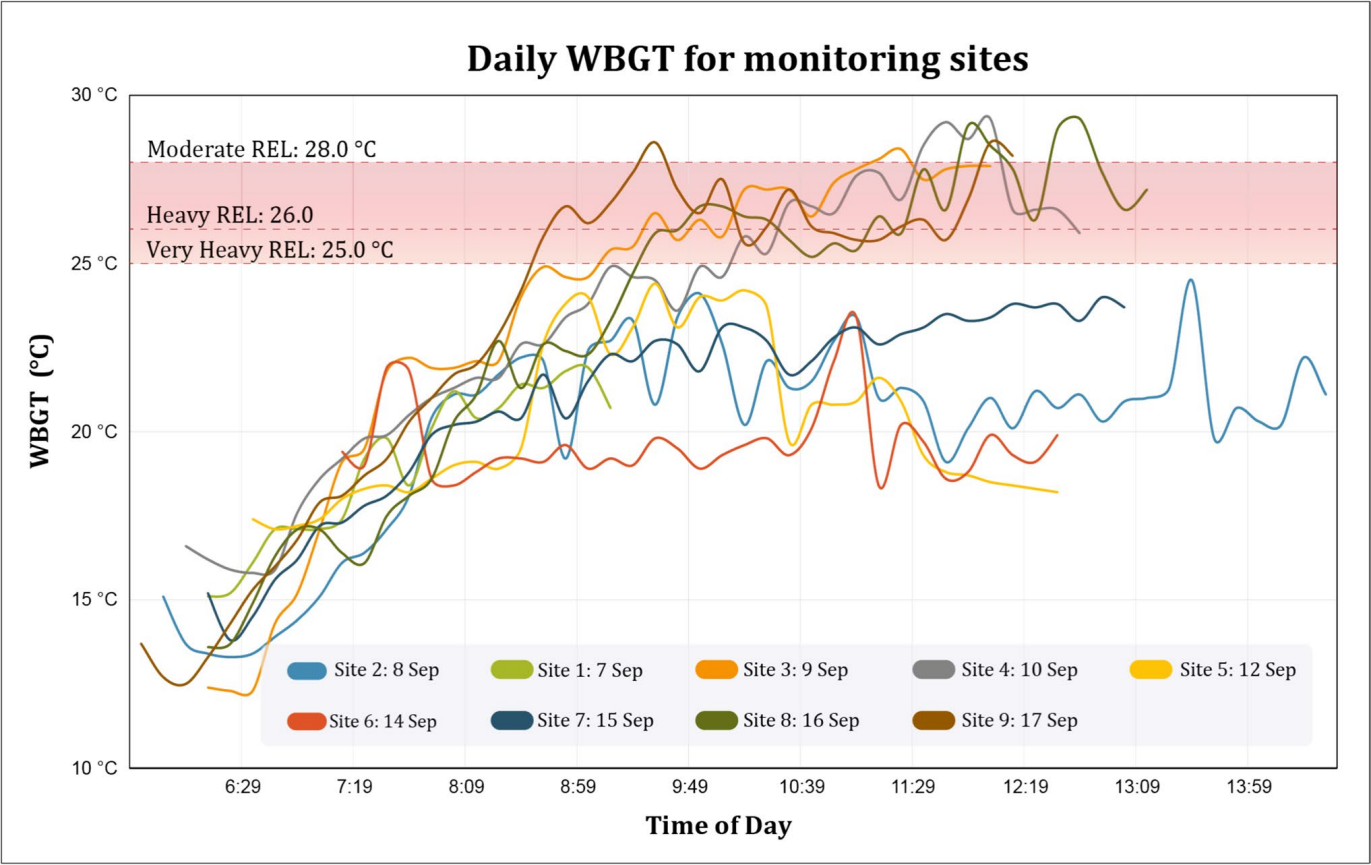
Daily WBGT at monitoring sites. The recommended exposure limits (REL; NIOSH 2016) for moderate, heavy, and very heavy workload are illustrated

Figure 3 presents composite results of the nine sites where WBGT was monitored for sugarcane cutters and pesticide applicators. Overall, the earliest observed starting time was 0550 h, and the latest monitoring time was 1430 h. Monitoring in some sites (e.g., site 2) ended much earlier because that day, only pesticide applicator groups were working and finished their task early, unlike most of the days when both sugarcane cutters and pesticide applicators were working. The measurements in all sites had an upward trend from the early hours, stabilizing from around 0800 h. On four days, the maximum WBGT was above the 25.0 °C NIOSH recommended exposure limit for very heavy workload. On four days it surpassed the 26.0 °C limit for heavy workload, indicating the need for regulated hourly rest periods.

Temperature, relative humidity, and WBGT measurements at the nine sites are displayed in Table 1. For workers with a high workload (cane cutters), the observed daily average maximum WBGT of 28.6 °C was above the NIOSH recommended exposure limit (REL) of 26 °C... For pesticide applicators with moderate workload, ending their workday earlier than cutters, often between 0800 and 1030 h, had a lower daily average maximum WBGT of 25.6 °C, but with correction for the PPE clothing, their effective WBGT would become higher.

**Table 1:** Air temperature, relative humidity, and WBGT (uncorrected for clothing) in Eswatini sugar fields, September 2022

Variable	Air temperature mean & range (°C)		Relative humidity mean & range (%)		WBGT mean & range (°C)	
Location	Sugarcane cutting	Pesticide application	Sugarcane cutting	Pesticide application	Sugarcane cutting	Pesticide application
Site 1	23.9 14.2–29.4	22.1 14.2–29.4	50.2 34.5–89.2	59.3 34.5–89.2	19.9 13.3—24.5	19.0 13.3–24.1
Site 2	22.3 18.2–26.1	22.3 18.2–26.1	57.1 46.0–72.5	57.1 46.0–72.5	20.7 15.1–21.9	19.3 15.1–21.9
Site 3	28.5 13.1–35.9	26.3 13.1–34.7	45.3 27.0–91.3	50.8 30.9–91.3	23.3 12.3–28.4	21.8 12.3–27.2
Site 4	29.0 19.3–38.8	25.8 19.3–33.1	39.5 17.7–60.9	47.4 32.0–60.9	23.3 15.8–29.3	21.4 15.8–26.8
Site 5	24.0 18.5–31.6	24.5 18.5–31.6	59.1 40.1–81.7	60.0 40.1–81.7	20.3 17.1–24.4	20.6 17.1–24.4
Site 6	22.1 20.4–26.6	21.5 20.4–25.3	66.4 52.2–74.2	70.4 63.7–74.2	19.6 18.4–23.4	19.4 18.4–21.9
Site 7	24.4 15.7–29.3	22.4 15.7–27.3	51.4 35.6–81.0	58.4 42.7–81.0	21.0 13.8–24.0	19.7 13.8–23.1
Site 8	28.5 15.4–38.8	24.7 15.4–33.5	41.4 19.4–77.3	50.7 28.8–77.3	23.4 13.6–29.3	21.2 13.6–26.7
Site 9	27.2 13.7–36.6	25.2 13.7–36.3	48.1 24.4–91.8	54.8 30.2–91.8	22.7 12.5–28.6	21.4 12.5–28.6
Overall (median & range)	28.5 23.9–29.0	24.0 22.1–29.0	45.3 39.5–51.2	51.2 39.5–66.4	23.3 19.9–23.4	20.7 19.6–23.4

### Study participants and characteristics

All participants were male. All applicators and most cutters were Eswatini nationals. A majority were aged 30 – 54 years (75%), and a few (7%) participants were over 54 years, with 2 participants ≥ 70 years (Table 2). Most cutters (70%) reported physically demanding work 7 days a week, while most applicators reported 6 days a week. The reported water intake during the workday ranged from 0–5 L, with 1.4 L as a mean.

**Table 2:** Sociodemographic, work and lifestyle characteristics of sugarcane workers in Eswatini

Characteristic	Categories	Sugarcane cutter	Pesticide applicator	Total
		N = 267 (%)	N = 125 (%)	392 (%)
Age (years)	Mean (SD; Range)	36.1 (11.6; 18–79)	33.3 (10.3; 19–70)	35.0 (11.3; 18–79)
Height (cm)	Mean (SD; Range)	163.1 (9.1; 140–198)	162.0 (9.0; 145–199)	163.0 (9.1; 140–199)
Weight (kg)	Mean (SD; Range)	67.4 (9.1; 48–119)	69.8 (9.4; 52–99)	68.2 (9.3; 48–119)
Body Mass Index (kg/m^2^)	Mean (SD; Range)	25.2 (3.1; 16.0–39.3)	26.7 (3.7; 16.1–38.5)	25.7 (3.4; 16.0–39.3)
Language	SiSwati	205 (76.8%)	125 (100%)	330 (84.2%)
	English	2 (0.7%)	0 (0%)	2 (0.5%)
	Portuguese	60 (22.5%)	0 (0%)	60 (15.3%)
Nationality	Eswatini	203 (76.0%)	125 (100%)	328 (83.7%)
	Mozambique	60 (22.5%)	0 (0%)	60 (15.3%)
	Malawi	3 (1.1%)	0 (0%)	3 (0.7%)
	Zimbabwe	1 (0.4%)	0 (0%)	1 (0.3%)
Workdays per week (days)	Mean (Range; SD)	6.5 (3–7; 0.9)	6.0 (5–7; 0.2)	6.3 (3–7; 0.8)
Physically demanding work days	< 6 days/week	39 (14.6%)	10 (8.0%)	49 (12.5%)
	≥ 6 days/week	228 (85.4%)	115 (92.0%)	343 (87.5%)
Water Intake (litres)	Mean (Range; SD)	1.5 (0–5; 0.9)	1.2 (0–5; 0.8)	1.4 (0–5; 0.9)
Work Water Source	Surface	105 (39.8%)	79 (63.2%)	184 (47.3%)
	Tap water	143 (54.2%)	37 (29.6%)	180 (46.3%)
	Borehole/unprotected	16 (6.1%)	9 (7.2%)	25 (6.4%)
Non-steroidal anti-inflammatory drugs^1^	Yes	29 (10.9%)	11 (8.8%)	40 (10.2%)
Alcohol	Yes	124 (46.4%)	53 (42.4%)	177 (45.1%)
Sodas	Yes	256 (95.9%)	118 (94.4%)	374 (95.4%)
Energy Drinks	Yes	152 (56.9%)	80 (64.0%)	232 (59.2%)

^1^The question was: “Have you taken any pain medication for two weeks or longer in the last 3 months?”

### Workload assessment

Sugarcane cutters' QEC average scores for exposure of the back were very high and high for the wrist/hand, shoulder/arm, and neck. Exposure to other factors was moderate for work pace, and high for stress (Table 3 and Supplementary material 4). Observations of the sugarcane cutters' work were characterized by moderate to excessive flexing of the back. As the sugarcane cutters carried out their task, there were frequent (around 8 times per minute) to very frequent (around 12 times per minute and above) movements (Fig. 1). The workers' shoulders/arms, mostly at their waist height, were moving frequently to very frequently with some pauses. They mostly kept deviated wrists, with similar repeated motion patterns about 11 to more than 20 times per minute. Their necks twisted, occasionally to continuously, as they performed their task. In summary, the QEC observations, supported by the expert observation (BS), rated the overall workload for the cutters as high to very high.

**Table 3:** Level of exposure to physical work stressors for sugarcane workers in Eswatini, according to QEC structured observations. The total score for each body area is determined based on posture, load or force, duration, and frequency of movements.

Job Category	Exposure Area					
	Back	Shoulder/Arm	Wrist/Hand	Neck	Work Pace	Stress
Pesticide Applicator	27.3	27.3	28.0	8.0	1.0	4.7
	Moderate	Moderate	Moderate	Moderate	Low	Moderate
Sugarcane Cutter	41.3	36.0	35.3	14.7	4.7	12.0
	Very High	High	High	High	Moderate	High

Note: The numbers are sum of exposure scores averages for three observer groups per job category with corresponding QEC interpretation. (See also Supplementary material 4)

The amount of cane cut (length of row) over the day was available for 203 out of 267 cutters. The mean distance cut was 95.7 m (range 40 – 235). While all the sugarcane cutters were given the same quota of distance to cut at the beginning of the shift, some of those who finished their quota early provided help to their team members to finish. Other cutters were not assisted but worked from the start until they finished. Thus, the distance in meters (work output) is not equal to work intensity but rather a measure of productivity, as the duration of cutting varied between individuals.

The pesticide applicators' level of exposure to physical work stressors was moderate for the back, shoulders/arms, wrist/hand, and neck (Table 3). Exposure scores for work pace were low and moderate for stress. Carrying the spraying backpacks that were filled up to 16–20 L was reported to be the most physically demanding aspect. There were frequent flexing movements (around 8 times per minute) of the back as they performed their task, especially while they prepared the pesticide solution and when spraying in the fields (Fig. 2). The workers' shoulders/arms were mostly positioned below waist height. When moving the pump lever and nozzle, the wrists were mostly deviated with motions repeated about 11 to 20 times per minute.

The applicators´ workload was classified as moderate, based on the results of the QEC, the expert rating (BS), and the HR measurements. Compared to the cutters, the applicators' work had limited variability, as the pesticide applicators worked in teams, starting and finishing work together.

### Assessment of workload variability and estimated core temperature in pesticide workers

**Fig. 4:**
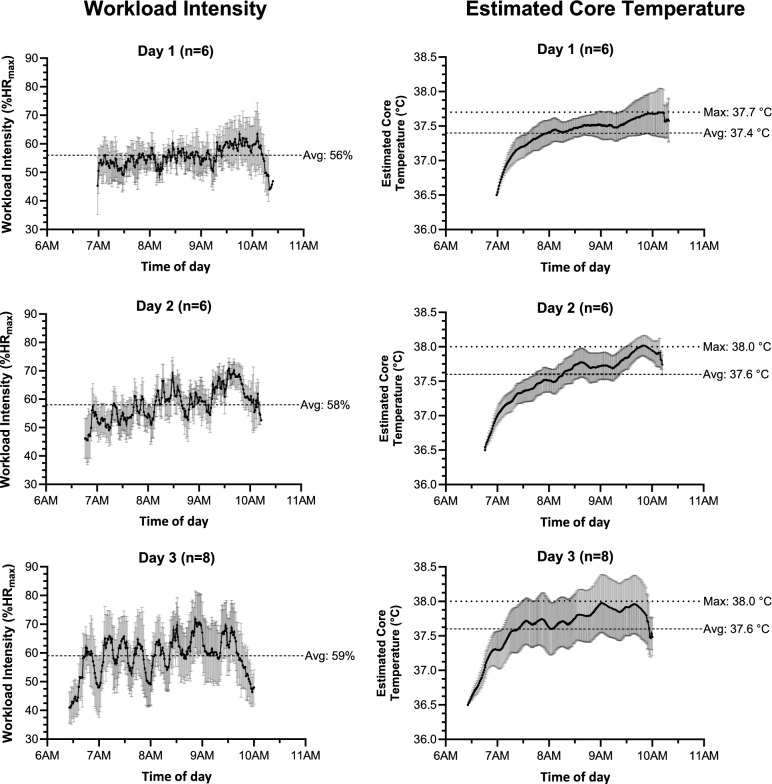
Rest pattern and average workload intensity (%HR_max_) (left panel) and estimated core temperature (ECTemp) (right panel) across the work shift for pesticide applicators during three observed workdays. Error bars indicate standard deviation. Dashed (-—-) and dotted (· · ·) lines indicate group averages and maximums, respectively. Note: estimated core temperature was calculated from sequential heart rate measurements.

On the three workdays where assessments of physiological workload were conducted, the average WBGT during the work shift was 28.2 ± 1.7 °C, 27.1 ± 2.5 °C, and 28.2 ± 2.1 °C. Maximum recorded WBGT was 30.6 °C, 32.1 °C, and 32.5 °C for the three days. The average work shift duration over the three observed workdays was 03:31 ± 00:12 h. Throughout their shift, workers stopped to refill their spraying containers as a group, with their workload intensity cyclically rising and falling across the day (most apparent at a group level on Day 3 of workload assessments; Fig. 4). The average %HR_max_ was 58 ± 6% for pesticide applicators with 9% of the work shift spent working at or above a hard intensity and 19% at a light intensity. The largest proportions of the work shift were spent at light-moderate (43%) and moderate (29%) workload intensities.

On average, maximum ECTemp for pesticide applicators during their work shift was 37.9 ± 0.4 °C (Fig. 4), while the proportion of the work shift spent at an ECTemp > 38.0 °C was 11 ± 19%. Nine of the 20 pesticide applicators exceeded an ECTemp of 38 °C during their shift, with one person exceeding 38 °C for 77% of his shift. Notably, the short, informal rest breaks with no shade when refilling spraying containers did not prevent ECTemp from rising continuously over the work shift.

### Pesticide exposure

None of the sugarcane cutters reported any concurrent pesticide work. However, 4 (1.5%) of the cutters reported that they sometimes worked in recently pesticide-sprayed fields.

Of all the applicators, 98 (78%) engaged in mixing pesticide solution, 109 (87%) loaded pesticide solution, and all except one supervisor applied pesticides in the fields. Most applicators reported use of Ametryn® (74.4%) and Bifenthrin® (68.0%), which are moderately hazardous pesticides according to the WHO classification of pesticides by hazard approach. Dicamba®, 2–4-D®, Flumioxasin®, Atrazine® and glyphosate were also frequently reported (Supplementary material 5). None of the pesticides reported were classified as extremely or highly hazardous.

Table 4 presents the self-reported personal protection safety practices for pesticide applicators. Few applicators had access to appropriate fully protective equipment. Specifically, a high percentage did not use air respirators, face masks or face shields, chemical overalls, or spray suits. However, filter cartridge respirators, chemical gloves, and safety shoes (rubber boots) were commonly used. They also had long plastic aprons covering the front and back (Fig. 2). Almost all pesticide applicators reported showering immediately after finishing work with pesticides before leaving their workplace. Only a few workers (1.6%) did not adhere to this practice.

**Table 4:** Use of personal protective equipment among 124 active pesticide applicators in Eswathini sugarcane fields

Practice/PPE	Use; n (%)
Air respirator	5 (4%)
General gloves	21 (17%)
Chemical overall	26 (21%)
Face masks	34 (27%)
Face shield	35 (28%)
Plastic overall	40 (32%)
Spray suit	44 (35%)
Eye protection	56 (45%)
Cloth overall	99 (80%)
Filter cartridge respirator	114 (92%)
Chemical gloves	116 (94%)
Shower after work	122 (98%)
Safety shoes	124 (100%)

## Discussion

Sugarcane cutters in Eswatini, working at small-scale farms hired by subcontractors, were frequently exposed to high ambient heat stress often exceeding RELs for heavy work. They had no access to regulated rest breaks, shade, hydration, and sanitation. Pesticide applicators were directly employed, had a moderate workload, and terminated their work before the hottest hours of the day. The personal protection equipment provided by the employer was limited and did not give full chemical protection while still adding substantially to heat stress.

Our observations on high workload and heat stress during manual sugarcane cutting are similar to reports from large mills in Central America and Thailand (Bodin et al. 2016; Boonruksa et al. 2020; Crowe et al. 2013; Dally et al. 2018; Glaser et al. 2022). A moderate physical workload has previously been reported in backpack pesticide applicators using indirect measures of workload (Crowe et al. 2010). In the current study, direct measures of workload indicated a light-moderate workload intensity across their shift (including rest breaks; 58%HR_max_), but still sufficient to result in ECTs exceeding 38 °C. Their impermeable PPE hinders heat dissipation, and clothing factors should be added when calculating the effective WBGT (ISO 1989).

During four of the nine days of measurements, the WBGT was above the REL for heavy work already at around 0900 h. A rise of estimated core temperatures in the initial hours of the work shift, even before WBGT peaks, demonstrates how workers push themselves more in the early hours. Even when WBGT is lower, as the workers intensify their work to maximize productivity, they also increase the risk of heat stress to themselves. This pattern, repeatedly seen among sugarcane cutters (Bodin et al. 2016, Hansson et al., 2019) and among the present pesticide applicators, calls for regulated rests already early in the workday, especially when workers are paid by piece or by designed quota (ILO 2024). The organization of pesticide work allowed for repeated short breaks while backpacks were refilled, however, without access to shade. Thus, their core temperature increased steadily over the shift.

We classified workload on group basis, based on onsite observations and structured assessment of the ergonomic demands. On an individual basis, the workload will inevitably vary, and depend on physical capacity (notably, there were workers as old as in their 70 s), work pacing, and work intensity. Although there was a similar daily quota for workers, the actual length of the row cane cut (a direct measure of productivity) varied, as well as the duration of cutting. For work intensity assessment, we would have needed monitoring of physiological responses such as heart rate while observing time spent performing tasks (Lee et al. 2022), preferably in combination with self-reported data on their perception of workload, and stress levels (Oliv et al. 2019).

No designated drinking water was provided at any of the workplaces. The average 1.5 L of water consumed by sugarcane cutters during a workday of 6 h was far less than the recommended volume of at least 0.6 L per hour during hard work at WBGTs 25–29 °C (ILO 2024). For pesticide applicators, the amount was also low. This indicates that there is a risk of daily repeated dehydration, which, in addition to heat stress, is a risk factor for the development of acute and chronic kidney disease (Hansson et al. 2024a, 2024b; Sorensen et al. 2019).

The sugarcane cutters did not engage in direct pesticide handling. Still, indirect work-related pesticide exposure could occur, as it was observed that sugarcane cutters carried their maize drink in used pesticide containers. Also, the workers´ drinking water, in addition to what they brought from home, was taken directly from the field irrigation system. Surface water was another common source; thus there is a potential for pesticide contamination (Damalas et al. 2016).

The applicators handled pesticides when mixing the pesticide solution, filling the backpacks, and spraying. Other occupational studies have demonstrated similar arrangements (Hyland et al. 2024; Liem et al. 2021). One of the safety precautions that pesticide applicators regularly practiced was taking a shower after the end of work, which has been reported as a challenge elsewhere (Negatu et al. 2016). The applicators did not have access to proper PPE such as air respirators and chemical overalls. Similar lack of safe pesticide-handling practices is commonly reported (Lari et al. 2023; Staudacher et al. 2020) and will expose workers through the dermal and inhalation routes (Damalas et al. 2016). Notably, spraying in forward-walking lines in adjacent cane rows allows for exposure from co-workers; forward walking may also cause higher exposure than walking backwards (Cao et al. 2018; Msibi et al. 2021). Ensuring distance between applicators during spraying in the field and proper rinsing practices to prevent residues and pesticide drift are needed (Damalas et al. 2016; Msibi et al. 2021).

The QEC ratings of sugarcane cutters' levels of musculoskeletal exposures were very high for the back and high for the shoulder/arm, wrist/hand, and neck. This indicates a high risk for musculoskeletal disorders. Systematic reviews and other literature have reported high risks of musculoskeletal disorders in sugarcane workers, as in many other agricultural workers (Akbar et al. 2023; Kee et al. 2019). While some studies among sugarcane workers reported high NSAIDs use, 25–50%, to relieve pain (García-Trabanino et al. 2015; Pundee et al. 2020), our findings are in line with other studies reporting limited use among sugarcane cutters (Glaser et al. 2020) and with no difference between cutters and applicators.

## Strengths and limitations

To our knowledge, this study is the first to describe work practices and occupational exposures among subcontracted sugarcane labourers working at small-holder farms. The relevance of this study also comes from its inclusive approach of including seasonal and migrant workers, a population vulnerable to occupational exposures (Alemu Gelaye et al. 2021). Rich contextual information provides valuable information for investigations of the health consequences of work and informs interventions to prevent heat stress.

A limitation of this observational study is that data was collected only over two weeks in September, and one week in November. While the work procedures are similar over the harvest, the short timescale cannot capture the variability of the climate over the harvest season. Given that the higher levels of heat during the summer, spanning from October to April, were still approaching when this study was done, higher heat stress levels are expected during the remaining part of the harvest season.

Workload was characterized on group (job) level. We could perform high-quality HR measurements on a subset of 20 pesticide applicators, but for the sugarcane cutters we relied on observations. The expert´s assessment, based on long experience from HR measurements in Central American sugarcane workers with similar cutting techniques and similar climate, strengthens the validity of the characterization for cane cutters.

Some of the migrant cane cutters did not understand the SiSwati local language. This challenge was resolved by identifying one Portuguese translator and an English speaker sourced from the study participants. The translators also had a good understanding of the local language. Hence, they were able to interpret the language of the migrant workers into the SiSwati language; thus, we believe there was little risk of misunderstanding.

## Conclusions and implications

Rising temperatures in the Sub-Saharan region (IPCC 2022) brought on by climate change pose a threat to the sugarcane industry because of the potential for extreme weather events and water shortages, which could result in lower yields (Ncoyini-Manciya et al. 2024; Ngcobo et al. 2023). Similarly, workers are susceptible to heat stress, affecting productivity and health. Proactive steps must be taken to safeguard the workers and modify farming methods to boost long-term agricultural productivity by ensuring the sustainability of crop production as well as the well-being of the workforce (Amoadu et al. 2023; Boonruksa et al. 2020). The manual cane harvesting on the smallholder farms in Eswatini is based on subcontracting of temporarily employed residential laborers, within-country migrants, and migrants from neighboring countries. This makes the workforce even more vulnerable (Min et al. 2013). Moreover, subcontracting obscures the occupational health and safety responsibilities between farmers, cooperatives, and subcontractors unless contractual agreements are transparent.

Minimising potential health risks due to heat stress and workload for manual sugarcane workers requires the implementation of a rest-shade-hydration intervention scheme. Such efforts have shown positive results in other sugarcane harvest settings, increasing productivity and with a positive return on investment (Bodin et al. 2016; Hansson et al. 2024a; Schlader et al. 2025). It involves compulsory 10–15 min hourly breaks in natural shade or tents from the onset of work, and enough drinking water supply close to the working area for workers to take about 0.6–0.8 L per hour (Glaser et al. 2022; Hansson et al. 2024b; ILO 2024; NIOSH 2016). Additionally, workers should be trained on the prevention of heat stress, particularly the concept of acclimatization to promote workers' gradual adaptation to working in hot environments (ILO 2024). When lightweight and loose-fitting clothing is not possible, as in pesticide application, the extra heat stress induced by the PPE must be taken into account. For pesticide applicators, examples of low-technology interventions to reduce pesticide exposure are the provision of regularly scheduled training` sessions on best spraying technique, access to PPE and training on its use and maintenance, encouraging experienced applicators to mentor others, and recognise and reward proper PPE use. Moreover, daily risk assessment of heat stress risk and awareness of heat strain symptoms by workers and field supervisors is important.

## Supplementary Information

Below is the link to the electronic supplementary material.

Supplementary file1 (DOCX 327 KB)

## Data Availability

The authors confirm that the data supporting this study are available and will be shared upon reasonable request through the corresponding author.

## References

[CR1] ACGIH (2007) Heat stress and strain: TLV physical agents documentation. American conference of Governmental Industrial Hygienists

[CR2] Ainembabazi, J. (2018). The 2015–16 El Niño-induced drought crisis in Southern Africa: What do we learn from historical data? 10th International Conference of Agricultural Economists.

[CR3] Akbar KA, Try P, Viwattanakulvanid P, Kallawicha K (2023) Work-related musculoskeletal disorders among farmers in the southeast Asia region: a systematic review. Saf Health Work 14(3):243–249 37818214 10.1016/j.shaw.2023.05.001 PMC10562125

[CR4] Alemu Gelaye K, Debalke G, Awoke Ayele T, Fekadu Wolde H, Sisay MM, Teshome DF, Akalu TY, Daba Wami S (2021) Occupational health problems among seasonal and migrant farmworkers in Ethiopia: a cross-sectional study. Risk Manag Healthcare Policy 14:4447–4456 10.2147/RMHP.S323503 PMC8566000 34744466

[CR5] Amoadu M, Ansah EW, Sarfo JO, Hormenu T (2023) Impact of climate change and heat stress on workers’ health and productivity: a scoping review. J Climate Change Health 12:100249

[CR6] Bernard TE (2014) Occupational heat stress In USA: whither we go? Ind Health 52(1):1–4 24531131 10.2486/indhealth.100 PMC4202768

[CR7] Bodin T, García-Trabanino R, Weiss I, Jarquín E, Glaser J, Jakobsson K, Lucas R, Wesseling C, Hogstedt C, Wegman D (2016) Intervention to reduce heat stress and improve efficiency among sugarcane workers in El Salvador: phase 1. Occup Environ Med 73(6):409–416 27073211 10.1136/oemed-2016-103555 PMC4893112

[CR8] Boonruksa P, Maturachon T, Kongtip P, Woskie S (2020) Heat stress, physiological response, and heat-related symptoms among Thai sugarcane workers. Int J Environ Res Public Health 17(17):6363 32882881 10.3390/ijerph17176363 PMC7503547

[CR9] Buller MJ, Tharion WJ, Cheuvront SN, Montain SJ, Kenefick RW, Castellani J, Latzka WA, Roberts WS, Richter M, Jenkins OC (2013) Estimation of human core temperature from sequential heart rate observations. Physiol Meas 34(7):781 23780514 10.1088/0967-3334/34/7/781

[CR10] Cao L, Zhang H, Li F, Zhou Z, Wang W, Ma D, Yang L, Zhou P, Huang Q (2018) Potential dermal and inhalation exposure to imidacloprid and risk assessment among applicators during treatment in cotton field in China. Sci Total Environ 624:1195–1201 29929232 10.1016/j.scitotenv.2017.12.238

[CR11] Cockburn J, Coetzee H, Van den Berg J, Conlong D, Witthöft J (2014) Exploring the role of sugarcane in small-scale farmers’ livelihoods in the Noodsberg area, KwaZulu-Natal, South Africa. S Afr J Agricu Ext 42(1):80–97

[CR12] Coco A, Jacklitsch B, Williams J, Kim J-H, Musolin K, Turner N (2016) Criteria for a recommended standard: occupational exposure to heat and hot environments. DHHS (NIOSH) Publication

[CR13] Crowe J, Manuel Moya-Bonilla J, Román-Solano B, Robles-Ramírez A (2010) Heat exposure in sugarcane workers in Costa Rica during the non-harvest season. Glob Health Action 3(1):5619 10.3402/gha.v3i0.5619 PMC2998054 21139704

[CR14] Crowe J, Wesseling C, Solano BR, Umaña MP, Ramírez AR, Kjellstrom T, Morales D, Nilsson M (2013) Heat exposure in sugarcane harvesters in Costa Rica. Am J Ind Med 56(10):1157–1164 23775893 10.1002/ajim.22204

[CR15] Crowe J, Knechtle B, Rojas-Valverde D (2023) Acute and long-term health issues of occupational exposure to heat and high physical loads (Vol. 14). Frontiers Media SA, p 1304229 10.3389/fphys.2023.1304229 PMC10599243 37885798

[CR16] Dally M, Butler-Dawson J, Krisher L, Monaghan A, Weitzenkamp D, Sorensen C, Johnson RJ, Carlton EJ, Asensio C, Tenney L (2018) The impact of heat and impaired kidney function on productivity of Guatemalan sugarcane workers. PLoS ONE 13(10):e0205181 30289894 10.1371/journal.pone.0205181 PMC6173423

[CR17] Damalas CA, Koutroubas SD (2016) Farmers’ exposure to pesticides: toxicity types and ways of prevention (Vol. 4). MDPI, p 1 10.3390/toxics4010001 PMC5606636 29051407

[CR18] David G, Woods V, Li G, Buckle P (2008) The development of the Quick Exposure Check (QEC) for assessing exposure to risk factors for work-related musculoskeletal disorders. Appl Ergon 39(1):57–69 17512492 10.1016/j.apergo.2007.03.002

[CR19] Ekiti ME, Zambo J-B, Assah FK, Agbor VN, Kekay K, Ashuntantang G (2018) Chronic kidney disease in sugarcane workers in Cameroon: a cross-sectional study. BMC Nephrol 19:1–8 29334929 10.1186/s12882-017-0798-9 PMC5769452

[CR20] ESA. (2023). Eswatini Sugar Association (ESA): Cane production statistics. Retrieved 31 July 2024 from https://esa.co.sz/cane-production-statistics/

[CR21] ESNAU. (2023). Membership. https://www.esnau.co.sz/membership/

[CR22] Eswatini. (2022). State of the climate. http://www.swazimet.gov.sz/ADVISORY/NEW_BULLETINS/state_of_the_climate.pdf

[CR23] Flouris AD, Dinas PC, Ioannou LG, Nybo L, Havenith G, Kenny GP, Kjellstrom T (2018) Workers’ health and productivity under occupational heat strain: a systematic review and meta-analysis. Lancet Planet Health 2(12):e521–e531 30526938 10.1016/S2542-5196(18)30237-7

[CR24] García-Trabanino R, Jarquín E, Wesseling C, Johnson RJ, González-Quiroz M, Weiss I, Glaser J, Vindell JJ, Stockfelt L, Roncal C (2015) Heat stress, dehydration, and kidney function in sugarcane cutters in El Salvador–a cross-shift study of workers at risk of Mesoamerican nephropathy. Environ Res 142:746–755 26209462 10.1016/j.envres.2015.07.007

[CR25] Gibb K, Beckman S, Vergara XP, Heinzerling A, Harrison R (2024) Extreme heat and occupational health risks. Annu Rev Public Health 45:315–335 38166501 10.1146/annurev-publhealth-060222-034715

[CR26] Glaser J, Hansson E, Weiss I, Wesseling C, Jakobsson K, Ekström U, Apelqvist J, Lucas R, Monge EA, Peraza S (2020) Preventing kidney injury among sugarcane workers: promising evidence from enhanced workplace interventions. Occup Environ Med 77(8):527–534 32404530 10.1136/oemed-2020-106406 PMC7402461

[CR27] Glaser J, Wegman DH, Arias-Monge E, Pacheco-Zenteno F, Prince H, Chavarria D, Martinez-Cuadra WJ, Jakobsson K, Hansson E, Lucas RA (2022) Workplace intervention for heat stress: essential elements of design, implementation, and assessment. Int J Environ Res Public Health 19(7):3779 35409463 10.3390/ijerph19073779 PMC8998134

[CR28] H Hathaway M, L Patil C, Odhiambo A, Onyango D, Dorevitch S (2023) Prevalence and predictors of chronic kidney disease of undetermined causes (CKDu) in Western Kenya’s “sugar belt”: a cross-sectional study. BMC Nephrol 24(1):157 37280533 10.1186/s12882-023-03213-2 PMC10243037

[CR65] Hansson E, Glaser J, Weiss I, Ekström U, Apelqvist J, Hogstedt C, Peraza S, Lucas R, Jakobsson K, Wesseling C, Wegman DH (2019). Workload and cross-harvest kidney injury in a Nicaraguan sugarcane worker cohort. Occup Environ Med. 2019 Nov;76(11):818-826. 10.1136/oemed-2019-105986 PMC6839725 31611303

[CR29] Hansson E, Jakobsson K, Glaser J, Wesseling C, Chavarria D, Lucas RA, Prince H, Wegman DH (2024a) Impact of heat and a rest-shade-hydration intervention program on productivity of piece-paid industrial agricultural workers at risk of chronic kidney disease of nontraditional origin. Annal Work Expo Health 68(4):366–375 10.1093/annweh/wxae007 PMC11033565 38367206

[CR30] Hansson E, Jakobsson K, Glaser JR, Wesseling C, Chavarría D, Lucas RA, Wegman DH (2024b) Association between acute kidney injury hospital visits and environmental heat stress at a Nicaraguan sugarcane plantation. Workplace Health Safety 72(4):131–142 38591368 10.1177/21650799241235410 PMC11055406

[CR31] Hyland C, Meierotto L, Som Castellano RL, Curl CL (2024) Mixed-methods assessment of farmworkers’ perceptions of workplace compliance with worker protection standards and implications for risk perceptions and protective behaviors. J Agromed 29:1–18 10.1080/1059924X.2024.2307483 38284770

[CR32] ILO. (2024). Heat at Work: Implications for Safety and Health. A Global Review of the Science, Policy and Practice.

[CR33] IPCC. (2022). Contribution of Working Group II to the Sixth Assessment Report of the Intergovernmental Panel on Climate Change. In: H.-O. Pörtner, D.C. Roberts, M. Tignor, E.S. Poloczanska, K. Mintenbeck, A. Alegría, M. Craig, S. Langsdorf, S. Löschke, V. Möller, A. Okem, B. Rama (eds.). Cambridge University Press, Cambridge, UK and New York, NY, USA.

[CR34] ISO (1989) Hot Environments: Estimation of the Heat Stress on Working Man, Based on the WBGT-index (wet Bulb Globe Temperature). International Organization for Standardization

[CR35] Kaahwa RM, Oyet SM, Muggaga C, Okello-Uma I (2023) The influence of sugarcane growing by smallholder farmers on household livelihood, food security, and nutrition status of children below five years in mid-western Uganda. J Agric Food Res 14:100895

[CR36] Kee D, Haslam R (2019) Prevalence of work-related musculoskeletal disorders in agriculture workers in Korea and preventative interventions. Work 64(4):763–775 31815716 10.3233/WOR-193038

[CR37] Kiatkitroj K, Arphorn S, Tangtong C, Maruo SJ, Ishimaru T (2021) Risk factors associated with heat-related illness among sugarcane farmers in Thailand. Ind Health 60(5):447–458 34819408 10.2486/indhealth.2021-0161 PMC9539147

[CR38] Kjellström T, Maître N, Saget C, Otto M, Karimova T (2019) Working on a warmer planet: The impact of heat stress on labour productivity and decent work. ILO

[CR39] Lari S, Yamagani P, Pandiyan A, Vanka J, Naidu M, Senthil Kumar B, Jee B, Jonnalagadda PR (2023) The impact of the use of personal-protective-equipment on the minimization of effects of exposure to pesticides among farm-workers in India. Front Public Health 11:1075448 37026139 10.3389/fpubh.2023.1075448 PMC10072124

[CR40] Lee W, Lin J-H, Howard N, Bao S (2022) Methods for measuring physical workload among commercial cleaners: a scoping review. Int J Ind Ergon 90:103319

[CR41] Liem JF, Mansyur M, Soemarko DS, Kekalih A, Subekti I, Suyatna FD, Suryandari DA, Malik SG, Pangaribuan B (2021) Cumulative exposure characteristics of vegetable farmers exposed to Chlorpyrifos in Central Java-Indonesia; a cross-sectional study. BMC Public Health 21(1):1066 34090393 10.1186/s12889-021-11161-5 PMC8178818

[CR42] Lucas RA, Skinner BD, Arias-Monge E, Jakobsson K, Wesseling C, Weiss I, Poveda S, Cerda-Granados FI, Glaser J, Hansson E (2023) Targeting workload to ameliorate risk of heat stress in industrial sugarcane workers. Scand J Work Environ Health 49(1):43 36209512 10.5271/sjweh.4057 PMC10549916

[CR43] Medina Hidalgo D, Mallette A, Nadir S, Kumar S (2024) The future of the sugarcane industry in Fiji: climatic, non-climatic stressors, and opportunities for transformation. Front Sustain Food Syst 8:1358647

[CR44] Min KB, Park SG, Song JS, Yi KH, Jang TW, Min JY (2013) Subcontractors and increased risk for work-related diseases and absenteeism. Am J Ind Med 56(11):1296–1306 23794385 10.1002/ajim.22219

[CR45] Msibi SS, Chen CY, Chang CP, Chen CJ, Chiang SY, Wu KY (2021) High pesticide inhalation exposure from multiple spraying sources amongst applicators in Eswatini. S Afr Pest Manag Sci 77(10):4303–4312 10.1002/ps.6459 33942970

[CR46] Ncoyini-Manciya Z, Manciya S (2024) Validating small-scale sugarcane farmers’ climate perceptions through scientific climate data to enhance awareness of climate change: the case of Swayimana Area in KZN Midlands, South Africa. S Afr J Agric Ext 52(3):36–54

[CR47] Negatu B, Kromhout H, Mekonnen Y, Vermeulen R (2016) Use of chemical pesticides in Ethiopia: a cross-sectional comparative study on knowledge, attitude and practice of farmers and farm workers in three farming systems. Ann Occup Hyg 60(5):551–566 26847604 10.1093/annhyg/mew004

[CR48] Ngcobo S, Hill T, Jewitt G, Archer E (2023) A yield gap analysis to assess vulnerability of commercial sugarcane to climatic extremes in southern Africa. J Agric Food Res 14:100734

[CR49] Nhamo, G. (2017). An assessment of Swaziland sugarcane farmer associations’ vulnerability to climate change.

[CR50] Oliv S, Gustafsson E, Baloch AN, Hagberg M, Sandén H (2019) The quick exposure check (QEC)—Inter-rater reliability in total score and individual items. Appl Ergon 76:32–37 30642522 10.1016/j.apergo.2018.11.005

[CR51] NIOSH. (2016). NIOSH criteria for a recommended standard: occupational exposure to heat and hot environments. By Jacklitsch B, Williams WJ, Musolin K, Coca A, Kim J-H, Turner N. Cincinnati, OH: U.S. Department of Health and Human Services, Centers for Disease Control and Prevention, National Institute for Occupational Safety and Health*.*

[CR52] Phajan T, Nilvarangkul K, Settheetham D, Laohasiriwong W (2014) Work-related musculoskeletal disorders among sugarcane farmers in north-eastern Thailand. Asia Pac J Public Health 26(3):320–327 24658706 10.1177/1010539514528026

[CR53] Pundee R, Kongtip P, Nankongnab N, Anutrakulchai S, Robson MG, Woskie S (2020) Cross-shift change of acute kidney injury biomarkers in sugarcane farmers and cutters. Hum Ecol Risk Assess Int J 27(5):1170–1187 10.1080/10807039.2020.1812049 PMC8291722 34290492

[CR54] Radir, A. F., Hashim, Z., Phan, K., Sao, V., & Hashim, J. H. (2017). The impact of heat on health and productivity among sugarcane workers in Kampong Cham, Cambodia. Asia Pac Environ Occup Health J,* 3*(1).

[CR66] R Core Team. (2023). R: A Language and Environment for Statistical Computing_. R Foundation for Statistical Computing, Vienna, Austria. Retrieved 31 July 2024 from https://www.R-project.org/

[CR55] Ruths JC, Shikida PFA, Fracarolli IFL (2023) Rural work in the sugarcane sector and its influences on health: scoping review. Revista Brasileira De Medicina Do Trabalho 21(1):e2023779 37197339 10.47626/1679-4435-2023-779 PMC10185400

[CR56] Santos UP, Zanetta DMT, Terra-Filho M, Burdmann EA (2015) Burnt sugarcane harvesting is associated with acute renal dysfunction. Kidney Int 87(4):792–799 25229334 10.1038/ki.2014.306

[CR57] Schlader, Z., Boswell, T., Prince, H., Wesseling, C., Amorim, F., Arias, E., Poveda, S., Hansson, E., Lucas, R., & Jakobsson, K. (2025). A Rest-Shade-Hydration-Hygiene program reduces acute kidney injury and increases production at a sugar mill in Nicaragua, an economic analysis. *medRxiv*, 2025.2002. 2019.25322486.

[CR58] Sorensen CJ, Butler-Dawson J, Dally M, Krisher L, Griffin BR, Johnson RJ, Lemery J, Asensio C, Tenney L, Newman LS (2019) Risk factors and mechanisms underlying cross-shift decline in kidney function in Guatemalan sugarcane workers. J Occup Environ Med 61(3):239–250 30575695 10.1097/JOM.0000000000001529 PMC6416034

[CR59] Sorensen CJ, Krisher L, Butler-Dawson J, Dally M, Dexter L, Asensio C, Cruz A, Newman LS (2020) Workplace screening identifies clinically significant and potentially reversible kidney injury in heat-exposed sugarcane workers. Int J Environ Res Public Health 17(22):8552 33218070 10.3390/ijerph17228552 PMC7698805

[CR60] Staudacher P, Fuhrimann S, Farnham A, Mora AM, Atuhaire A, Niwagaba C, Stamm C, Eggen RI, Winkler MS (2020) Comparative analysis of pesticide use determinants among smallholder farmers from Costa Rica and Uganda. Environ Health Insights 14:1178630220972417 33402828 10.1177/1178630220972417 PMC7739084

[CR61] Tanaka H, Monahan KD, Seals DR (2001) Age-predicted maximal heart rate revisited. J Am Coll Cardiol 37(1):153–156 11153730 10.1016/s0735-1097(00)01054-8

[CR62] Tourism Mo, Affairs E. (2011). Swaziland's Second National Communication to the United Nations Framework Convention for Climate Change Final Report. In: MoTEA.

[CR63] Varotsos C, Sarlis NV, Mazei Y, Saldaev D, Efstathiou M (2024) A composite tool for forecasting El Niño: the case of the 2023–2024 event. Forecasting 6(1):187–203

[CR64] WHO (2020) The WHO recommended classification of pesticides by hazard and guidelines to classification 2019. World Health Organization

