# Supplementary material for: Work and work exposures in sugarcane farming in Eswatini, Southern Africa
Source: Int Arch Occup Environ Health. 2025 May 14;98(4-5):421–35. doi: 10.1007/s00420-025-02140-z (PMC12238168; doi:10.1007/s00420-025-02140-z)
Supplement: Supplementary file 1 — Supplementary file1 (DOCX 327 KB) [file 420_2025_2140_MOESM1_ESM.docx]

Supplementary information

**WORK AND WORK EXPOSURES IN SUGARCANE FARMING IN ESWATINI, SOUTHERN AFRICA**

Msibi SC^1, 2^, Naidoo S^1^, Jakobsson K^,5^, Glaser J^5^, Skinner B^5,6^, Naidoo RN^3^

**Affiliations:**

^1^ Discipline of Public Health Medicine, School of Nursing and Public Health, University of KwaZulu-Natal, Durban, South Africa.

^2^ Discipline of Public Health Management, Institute of Development Management, Manzini, Eswatini

^3^ Discipline of Occupational and Environmental Health, School of Nursing and Public Health, University of KwaZulu-Natal, Durban, South Africa

^4^ School of Public Health and Community Medicine, Institute of Medicine, University of Gothenburg, Gothenburg,

^5^ La Isla Network, 2219 California Ave NW, #52, Washington, DC 20008, USA.

^6^ School of Sport, Exercise and Rehabilitation Sciences, University of Birmingham, Birmingham, UK.

**CORRESPONDING AUTHOR**

Kristina Jakobsson, Occupational and Environmental Medicine, School of Public Health and Community Medicine, Institute of Medicine, Sahlgrenska Academy, University of Gothenburg, Gothenburg, Sweden. E-mail: kristina.jakobsson@amm.gu.se

**CONTENT**

1. Climatology
2. Specification of the WBGT measurements
3. Recommended exposure limits (REL)
4. QEC exposure scores
5. Reported use of pesticides
6. Questionnaire

1. Climatology


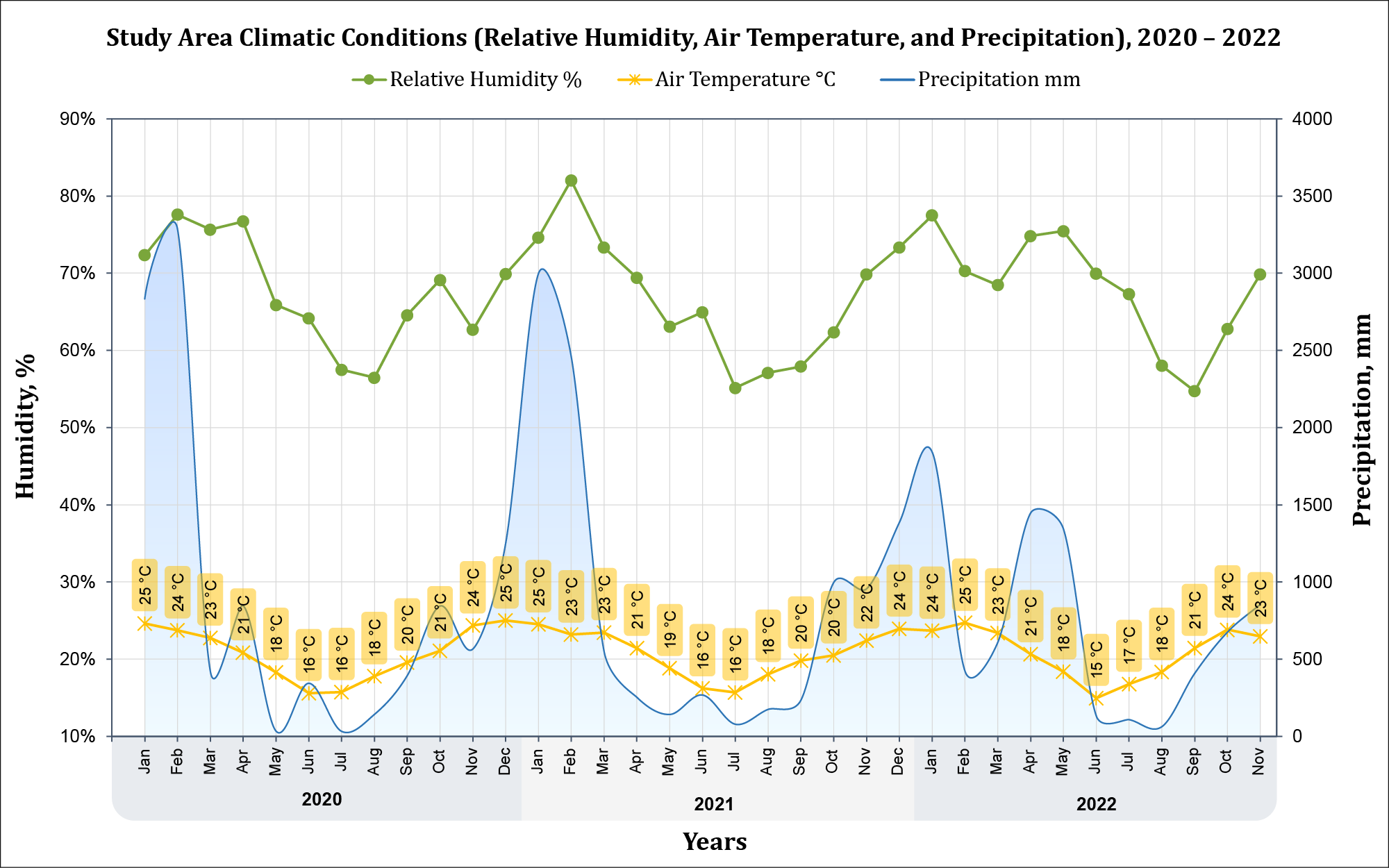


**Supplementary Figure 1**: Study area climatology: relative humidity, air temperature, and precipitation in Eswatini for the period 2020 – 2022

**Source:** Copernicus Climate Data Store. ERA5: Fifth generation of ECMWF atmospheric reanalyses of the global climate. Copernicus Climate Change Service Climate Data Store (CDS). Retrieved 15 February 2025.

1. Specification of the WBGT measurements

A portable AZ87786 WBGT data logger (AZ^®^) was used. The data logger specifications were defined as; WBGT range (0~50℃), global temperature accuracy outdoor (±1.5℃at 15~40℃, others ±2℃), humidity accuracy (±3%RH (at 25℃, 10~90%RH); others ±5%RH), air temperature accuracy ±0.6℃, and air temperature range (0~50℃) (Cooper et al., 2017). The supplier calibrated the recently purchased instrument and supplied a valid calibration certificate.

The data logger was placed for one day in nine of the twenty-three study sites, set in an outdoor mode to record air temperature, relative humidity, globe temperature, and WBGT. The nine sites selected were considered to be representative of all the twenty-three sites, which are located within the Lowveld agro-ecological zones of eSwatini with relatively similar altitudes, landforms, geology, soils, and vegetation (Swaziland, 2005). All farm sites where worker assessments were performed on a particular day were located within a radius of 7.74 km from the location of the data logger and at an altitude range of about 270 to 360 m above sea level.

At each of the nine sites, the data logger was placed on a tripod stand at a height of 2 meters from the ground, set to have a delay start time of 5 minutes to ensure that the instrument acclimatised to the ambient environmental conditions (Cooper et al., 2017). At each sampling site, the data logger was placed in field sections close to where workers had their shift for the day. Measurements were taken continuously from the beginning of each shift until all study participants had completed the assigned task for the day, with measurements recorded every 10 minutes. The earliest and latest measurements of the WBGT data logger were taken at 0549 h and 1429 h, respectively.

In total, 342 measurements with a sampling rate of 10 minutes were taken from nine sites over nine days of sampling. Overall, there was an estimated total of 62 hours and 24 minutes of sampling time, with an average of 6.9 hours per site and 38 measurements per site. Measurements were downloaded daily from the WBGT data logger to a laptop in Microsoft Excel CSV format files.

1. Recommended exposure limits (REL)

**National Institute of Occupational Safety and Health (NIOSH) WBGT recommended exposure limit for acclimatized workers (NIOSH, 2016)**

| **Workload Category** | **NIOSH REL** |
| --- | --- |
| Light | 30 °C |
| Moderate | 28 °C |
| Heavy | 26 °C |
| Very heavy | 25 °C |

**Note:** Workers not acclimated to the climate would expend more heat doing the same amount of labour in the same conditions.

1. QEC exposure scores

The total score for each body area was determined based on posture, load or force, duration, and frequency of movements. Exposure scores for the back, shoulder/arm, wrist/hand, and neck are categorised into four exposure categories: Low, Moderate, High, or Very High. (Robens Centre for Health Ergonomics, 1999)

**Important risk factors**

| **Back** | **Wrist/hand** |
| --- | --- |
| - Load weight - Duration - Frequency of movement - Posture | - Force - Duration - Frequency of movement - Posture |
| **Shoulder/arm** | **Neck** |
| - Load weight - Duration - Task height - Frequency of movement | - Duration - Posture - Visual demand |

**Exposure level**

| **Score** | **Low** | **Moderate** | **High** | **Very High** |
| --- | --- | --- | --- | --- |
| Back (static) | 8 - 15 | 16 - 22 | 23 - 29 | 29 - 40 |
| Back (moving) | 10 - 20 | 21 - 30 | 31 - 40 | 41 - 56 |
| Shoulder/arm | 10 - 20 | 21 - 30 | 31 - 40 | 41 - 56 |
| Wrist/hand | 10 - 20 | 21 - 30 | 31 - 40 | 41 - 56 |
| Neck | 4 - 6 | 8 - 10 | 12 - 14 | 16 - 18 |

1. Reported use of pesticides

**Supplementary Table:** Pesticides reported by pesticide applicators in sugarcane fields (n=125)

| **Brand Name** | **Active Ingredient(s)** | **Main Use** | **WHO Class** | **Responses**  **n (%)** |
| --- | --- | --- | --- | --- |
| Ametryn^®^ | Ametryn | Herbicide | II | 93 (74.4%) |
| Baseline^®^ | Bifenthrin | Insecticide | II | 85 (68.0%) |
| Di-plus^®^ | Dicamba | Herbicide | II | 43 (34.4%) |
|  | 2,4-D | Herbicide | II |  |
| Valtera^®^ | Flumioxazin | Herbicide | III | 37 (29.6%) |
| Atrazine^®^ | Atrazine | Herbicide | III | 36 (28.8%) |
| Glyphosate | Glyphosate | Herbicide | III | 31 (25.2%) |
| Paraquat | Paraquat | Herbicide | II | 16 (12.8%) |
| Springbok^®^ | Dimethenamid | Herbicide | II | 12 (9.6%) |
|  | Metazachlor | Herbicide | III |  |
| Pendimethalin | Pendimethalin | Herbicide | II | 9 (7.2%) |
| Cypermetrin | Cypermethrin | Insecticide | II | 8 (6.4%) |
| Trifluralin | Trifluralin | Herbicide | U | 6 (4.8%) |
| Acetochlor | Acetochlor | Herbicide | III | 5 (4.0%) |
| Triclopyr | Triclopyr | Herbicide | II | 5 (4.0%) |
| Tolla^®^ | Metolachlor | Herbicide | III | 3 (2.4%) |
|  | Benoxacor | Herbicide | U |  |
| Monosodium methylarsenate (MSMA) ^®^ | MSMA | Herbicide | Not specified | 2 (1.6%) |
| Kalach^®^ | Glyphosate | Herbicide | III | 1% |

***Note: Ia:*** *Extremely hazardous,* ***1b:*** *Highly hazardous,* ***II:*** *Moderately hazardous,* ***III:*** *Slightly hazardous,* ***U:*** *Unlikely to present acute hazard*

1. Questionnaire

**Sociodemographic, Health, Work and Pesticide Exposure Questionnaire**

| **Sociodemographic and Health Data** |
| --- |

1. Participant Code:____________________________
2. Body Mass Index: *Weight_____(kg) Height**_____(cm) Blood Pressure ____ (mmHG)*
3. Current place of residence: ____________________*(place)__________________(town)*
4. Home area *(permanent place of residence)*: ______________*(place)___________(town)*
5. Nationality: ___________________________
6. Race: *Black Coloured White*
7. Language: *SiSwati English Other (specify*):____________________
8. Do you have health insurance or medical aid? ______ *No ______ Yes*
9. What source of water do you use the most when you are at home?

*____Household water supply (piped) or Public tap/standpipe*

*____Borehole fitted with hand-pump*

*____Unprotected well*

*____Surface water (e.g., stream, river, lake, wetland)*

*____Tanker supplied water*

*____Bottled water*

1. What source of water do you use the most when you at work?

*____ Household water supply (piped) or Public tap/standpipe*

*____Borehole fitted with hand-pump*

*____Unprotected well*

*____Surface water (e.g., stream, river, lake, wetland)*

*____Tanker supplied water*

*____Bottled water*

1. Do you consume the following drinks?

*Coca-Cola:* *______ No ______ Yes*

*Pepsi: ______ No ______ Yes*

*Sprite:* *______ No ______ Yes*

*Red Bull: ______ No ______ Yes*

*Score: ______ No ______ Yes*

*Dragon: ______ No ______ Yes*

*Alcohol: ______ No ______ Yes*

1. How many cups of glasses of water did you drink yesterday? *(4 cups of water equivalent to 1L)* ________________________ *glasses of water*
2. Has a Doctor ever told you that you had been diagnosed with the following conditions?

*Kidney failure: ______ No ______ Yes*

*Kidney stones: ______ No ______ Yes*

*Pesticide poisoning: ______ No ______ Yes. Please Specify: _______________________*

1. Has any of your family or relatives ever been diagnosed with the following conditions?

*Kidney disease: ______ No ______ Yes*

*Kidney stones: ______* *No ______ Yes*

1. Have you taken any of the following pain medications for two weeks or longer? *(There is a catalogue with pictures of common pain medications that will help you identify these medications).*

In the last 3 months:

| **Medication** |  |
| --- | --- |
| Paracetamol (Panado) |  |
| Aspirin |  |
| Compral |  |
| Diclofenac (Diclo) |  |
| Ibuprofen (Brufen) |  |

In the last two years:

| **Medication** |  |
| --- | --- |
| Paracetamol (Panado) |  |
| Aspirin |  |
| Compral |  |
| Diclofenac (Diclo) |  |
| Ibuprofen (Brufen) |  |

1. In the last two weeks, have you experienced the following symptoms?

| ***Condition*** | ***No*** | ***Yes*** |
| --- | --- | --- |
| *Extremely dry mouth* |  |  |
| *Extremely thirsty* |  |  |
| *Dizziness* |  |  |
| *Feeling tired* |  |  |
| *Feeling exhausted* |  |  |
| *Not feeling well due to excessive sun exposure* |  |  |
| *Unusual salivation* |  |  |
| *Blurred vision* |  |  |
| *Sweating more than usual* |  |  |
| *Headache* |  |  |
| *Fever* |  |  |
| *Loss of appetite* |  |  |
| *Nausea* |  |  |
| *Vomiting* |  |  |
| *Rapid heartbeat / heart racing* |  |  |
| *Difficulty with balance* |  |  |
| *Confusion* |  |  |
| *Shaking or trembling of hands* |  |  |
| *Fainting* |  |  |
| *Muscle weakness* |  |  |
| *Cramps* |  |  |
| *Twitches of arms or legs* |  |  |
| *Pain when urinating* |  |  |
| *Very dark urine* |  |  |
| *Difficulty urinating* |  |  |
| *Tingling or needle prickle feeling (Paresthesia)* |  |  |

| **Work and Pesticide Exposure Date** |
| --- |

1. What is your current job title? ________________________
2. Please list five previous job titles you had in the past, excluding your current job title. Use the table below.

| **No.** | **Job Title** | **Industry** | **Location** | **Main Tasks** | **Start Year** | **End Year** |
| --- | --- | --- | --- | --- | --- | --- |
|  |  |  |  |  |  |  |
|  |  |  |  |  |  |  |
|  |  |  |  |  |  |  |
|  |  |  |  |  |  |  |
|  |  |  |  |  |  |  |

1. List the top three tasks that you do in your current job?

*a) ___________________________*

*b) ___________________________*

*c) ___________________________*

1. On a scale of 1 – 7 *(representative of the number of days in a week)*, how many days do you work per week?

1. On a scale of 1 – 7, how often do you do physically demanding tasks?

***___________ days(s)***

1. On a scale of 1 – 7, how often do you get exposed to working outside in the sunshine?

***___________ days(s)***

1. Do you personally do the following tasks? *[Yes / No – write in full words]*

*(a) Mixing pesticides: ___Yes ___No? ______ days*

*(b) Loading pesticides into spraying containers: ___Yes ___No? ______ days*

*(c) Spraying pesticides:* *___Yes ___No? ______ days*

1. Do you work in recently pesticide sprayed fields? *[Yes / No – write in full words]*

***Response: ______________***

***How many days per week, if you answered “Yes” ________________ days***

***If you answered “Yes” in the question above, please answer the following questions or stop here if you answered “No”.***

1. From the list of pesticides in the table below, please select all those that you have used since you started working in the sugarcane industry. *(There is a catalogue with pictures of pesticides that should help you identify these pesticides).*

| ***Pesticide Name*** |  |
| --- | --- |
| *Acetochlor* |  |
| *Atrazine* |  |
| *Imidacloprid* |  |
| *Cypermethrin* |  |
| *Diuron* |  |
| *Triclopyr* |  |
| *Glyphosate* |  |
| *Tebuthiuron* |  |
| *Diquat dibromide* |  |
| *Monosodium methylarsonate* |  |
| *Paraquat* |  |
| *Indoxacarb* |  |
| *Trifluralin* |  |
| *2,4D Phenoxy Comp* |  |

1. How many days per week do you mix, load or apply pesticides? _______*days*
2. What methods do you use when you apply pesticides in the fields? *(Select all methods you use or you have used).*

*(1) Backpack knapsack spraying*

*(2) Tractor spraying*

*(3) Vehicle mounted spraying*

1. Do you shower or bath after you have finished working with pesticides, before leaving work? *____Yes _____No*
2. Please select all types of personal protective wear that you use to mix, load or apply pesticides:

| ***PPE*** |  |
| --- | --- |
| *Safety shoes* |  |
| *Face shield* |  |
| *Eye protection* |  |
| *Face masks* |  |
| *Filter cartridge respirator* |  |
| *Air supplied respirator* |  |
| *Pesticide spray suit* |  |
| *Plastic type overall* |  |
| *Chemical type overall* |  |
| *Cloth type overall* |  |
| *General type gloves* |  |
| *Chemical type gloves* |  |
